# Case Report: Clinicopathological features and outcomes of superficial cervicovaginal myofibroblastoma: analysis of two cases and a review of the literature

**DOI:** 10.3389/fonc.2026.1819831

**Published:** 2026-05-13

**Authors:** Xiaoli Cai, Yanli Liu, Yunfeng Niu, Xiaodan Shen, Qian Zhang, Lei Liang, Shuang Liu

**Affiliations:** 1 Department of Pathology, Bethune International Peace Hospital, Shijiazhuang, Hebei, China; 2 Department of Ultrasound, Bethune International Peace Hospital, Shijiazhuang, Hebei, China; 3 Department of Gynecology, Bethune International Peace Hospital, Shijiazhuang, Hebei, China

**Keywords:** immunohistochemistry, molecular pathology, pathological diagnosis, prognosis, superficial cervicovaginal myofibroblastoma, vaginal tumor

## Abstract

**Background:**

Superficial cervicovaginal myofibroblastoma (SCVM) is a benign mesenchymal tumor that arises from the superficial stromal layer of the submucosal vagina and cervix in females. This tumor is clinically rare, occurs most frequently in women aged 20–60 years, and is often difficult to distinguish from other soft tissue tumors of the female genital tract due to overlapping clinical and pathological features.

**Case description:**

Two cases of SCVM located in the vagina of female patients were reported. One patient presented with vaginal bleeding, whereas the other lesion was identified during a routine physical examination in the absence of obvious clinical symptoms. Both patients underwent surgical resection of the tumor. The final diagnosis of SCVM was established through an integrated evaluation of histopathological morphology, immunohistochemical profiles, and molecular pathology findings. During follow-up periods ranging from 6 months to 1 year, no tumor recurrence or additional clinical symptoms were observed.

**Conclusion:**

A review of the English-language literature identified a total of 78 reported cases of SCVM, and their clinical manifestations, pathological features, and treatment outcomes were summarized. The findings indicate that SCVM occurs predominantly in women of reproductive age, demonstrates slow growth and benign biological behavior, exhibits hormone-related characteristics, and is associated with a favorable prognosis following surgical excision, with recurrence being extremely rare. Accurate diagnosis depends on the combined assessment of morphological features and immunohistochemical findings to reliably differentiate SCVM from other mesenchymal tumors of the cervix and vagina.

## 1 Introduction

Superficial cervicovaginal myofibroblastoma (SCVM) is an extremely rare benign mesenchymal tumor that primarily arises in the superficial stromal region of the female lower genital tract. Epidemiological data indicate that the global incidence of gynecologic tumors increased between 2019 and 2024, with benign tumors of the superficial lower genital tract accounting for approximately 15%–20% of all gynecologic tumors, whereas SCVM represents less than 0.1% of benign tumors (1). Since its initial description by Laskin et al. in 2001, only sporadic case reports have been documented. Owing to its very low incidence and the absence of specific clinical manifestations, limited clinical awareness has frequently led to misdiagnosis or overtreatment (2). With advances in precision medicine and continued improvement in the recognition and diagnostic approaches for rare gynecologic tumors, the number of reported SCVM cases has gradually increased, and diagnostic accuracy has improved through comprehensive assessment of histopathological morphology and immunohistochemical characteristics.

SCVM predominantly affects women aged 20–60 years. Clinical manifestations vary and may include irregular vaginal bleeding, increased vaginal discharge, or incidental findings in patients without obvious symptoms (3). From a morphological perspective, the tumor is characterized by proliferation of spindle-shaped myofibroblasts with bland nuclei and low mitotic activity. Immunohistochemical findings typically indicate positivity for smooth muscle actin (SMA) and desmin (DES), with possible expression of cluster of differentiation 34 (CD34), whereas S-100 protein is generally negative (4). In contrast to conventional smooth muscle tumors, SCVM lacks typical smooth muscle cell morphology and demonstrates greater sensitivity to estrogen. Surgical excision remains the primary treatment approach and is associated with a favorable prognosis and an extremely low rate of recurrence (5). However, due to the limited number of reported cases, current understanding of the biological behavior, molecular mechanisms, and long-term prognosis of this tumor remains incomplete. Therefore, detailed pathological analysis of such cases combined with a review of the literature was undertaken to provide additional references for clinical diagnosis and management and to enhance awareness of this rare tumor.

## 2 Case reports

### 2.1 Clinical data

One patient, aged 41 years, was admitted with intermittent irregular vaginal bleeding. The lesion was located in the posterior vaginal fornix and measured approximately 3.5 cm in diameter. On examination, the mass was firm, had a smooth surface, and bled easily on contact. The second patient was a 52-year-old perimenopausal woman admitted following incidental detection of a vaginal mass during a routine physical examination. This lesion was located on the posterior vaginal wall and measured approximately 2.5 cm in diameter. Neither patient reported additional symptoms, including abdominal pain or lower back pain. Both patients previously had regular menstrual cycles and good health. had no history of chronic diseases such as hypertension or diabetes, and reported no family history of tumors.

Gynecological examinations of both patients demonstrated normal vulvar development, a patent vagina, an anteverted uterus of normal size with good mobility, and no obvious abnormalities in the bilateral adnexal regions. ThinPrep cytologic testing indicated inflammatory changes without atypical cells, and human papillomavirus testing was negative in both patients. Gynecological ultrasonography demonstrated normal uterine size and morphology. A hypoechoic or isoechoic nodule with clear margins and heterogeneous internal echogenicity was identified on the posterior vaginal wall.

**Figure 1:**
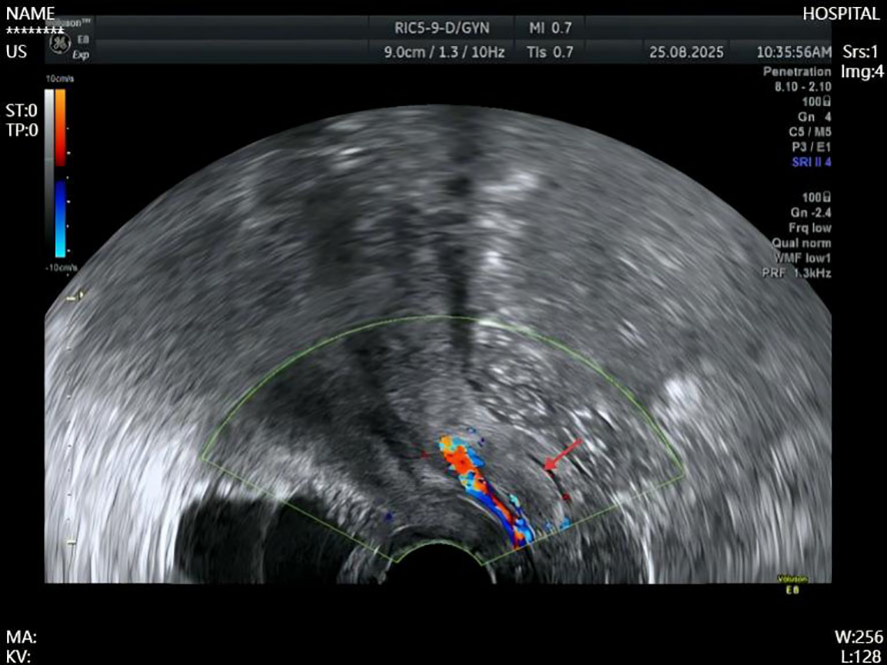
Transvaginal ultrasonographic findings in a 41-year-old patient with vaginal bleeding, demonstrating a 4.2 × 3.1 cm hypoechoic mass within the vagina, with blood flow signals within the lesion and along the posterior wall of the cervical canal. The red arrow indicates the tumor.

In the 41-year-old patient with vaginal bleeding, intratumoral blood flow signals were detected, along with blood flow in the posterior wall of the cervical canal, indicating a close anatomical relationship with the cervical canal (**Figure 1**). In both cases, a benign tumor of the cervix or vagina was initially considered, and surgical intervention was recommended.

### 2.2 Pathological examination

#### 2.2.1 Gross examination

**Figure 2:**
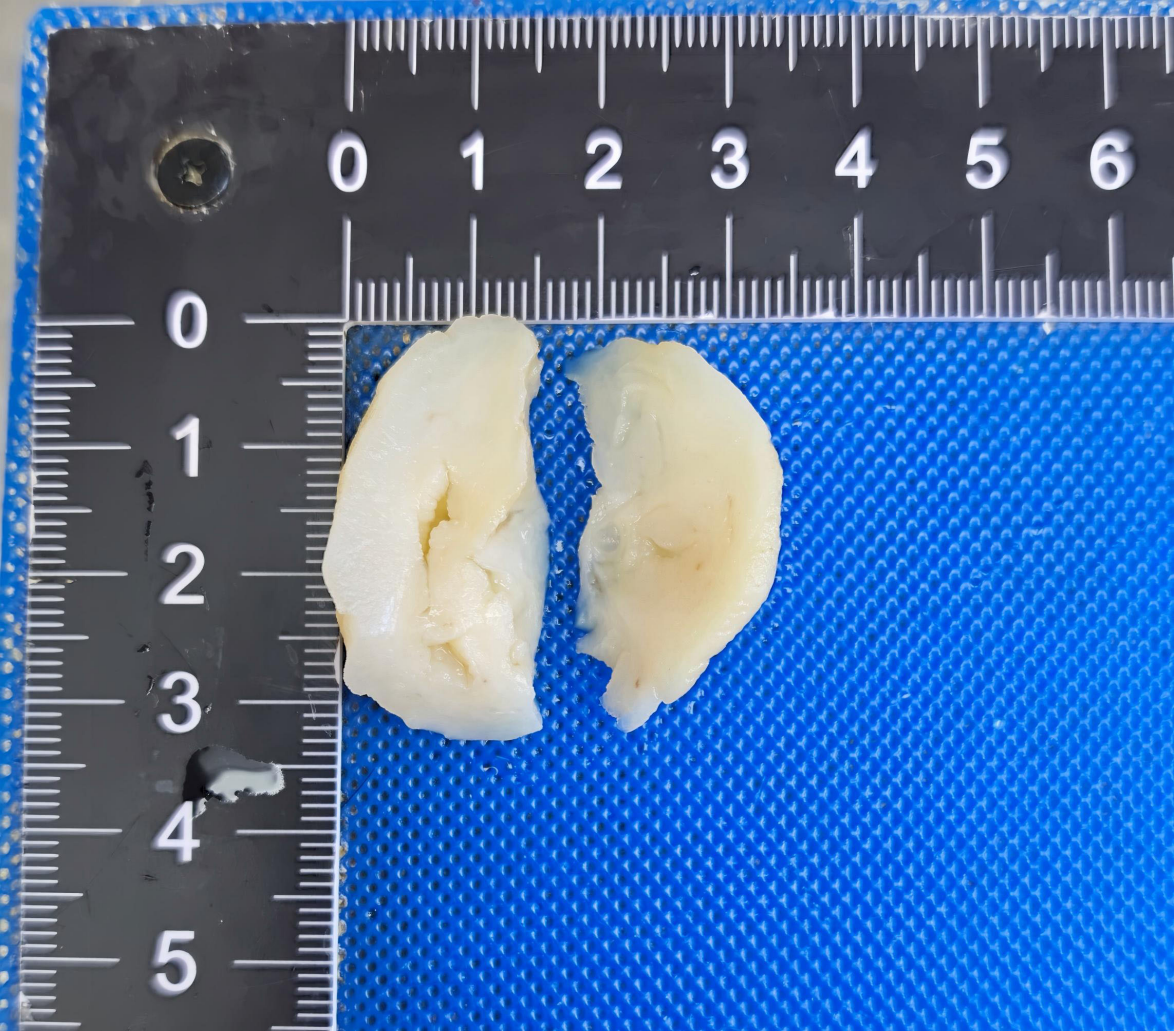
Gross morphology of superficial cervicovaginal myofibroblastoma demonstrating a nodular mass with a smooth surface. The cut surface is grayish-white to pale yellow, firm in consistency, and without obvious hemorrhage or necrosis. No distinct capsule is observed.

A nodular mass was identified on the posterior vaginal wall, with a smooth external surface. On cut surface, the lesion appeared grayish white to pale yellow, had a firm consistency, and demonstrated no obvious hemorrhage or necrosis. Although a well-defined capsule was absent, the tumor was relatively well demarcated from the surrounding normal vaginal stroma. No evidence of cystic degeneration or calcification was observed. The mass protruded into the vaginal lumen and exhibited a relatively smooth surface without apparent erosion or ulceration (**Figure 2**).

#### 2.2.2 Microscopic findings

**Figure 3:**
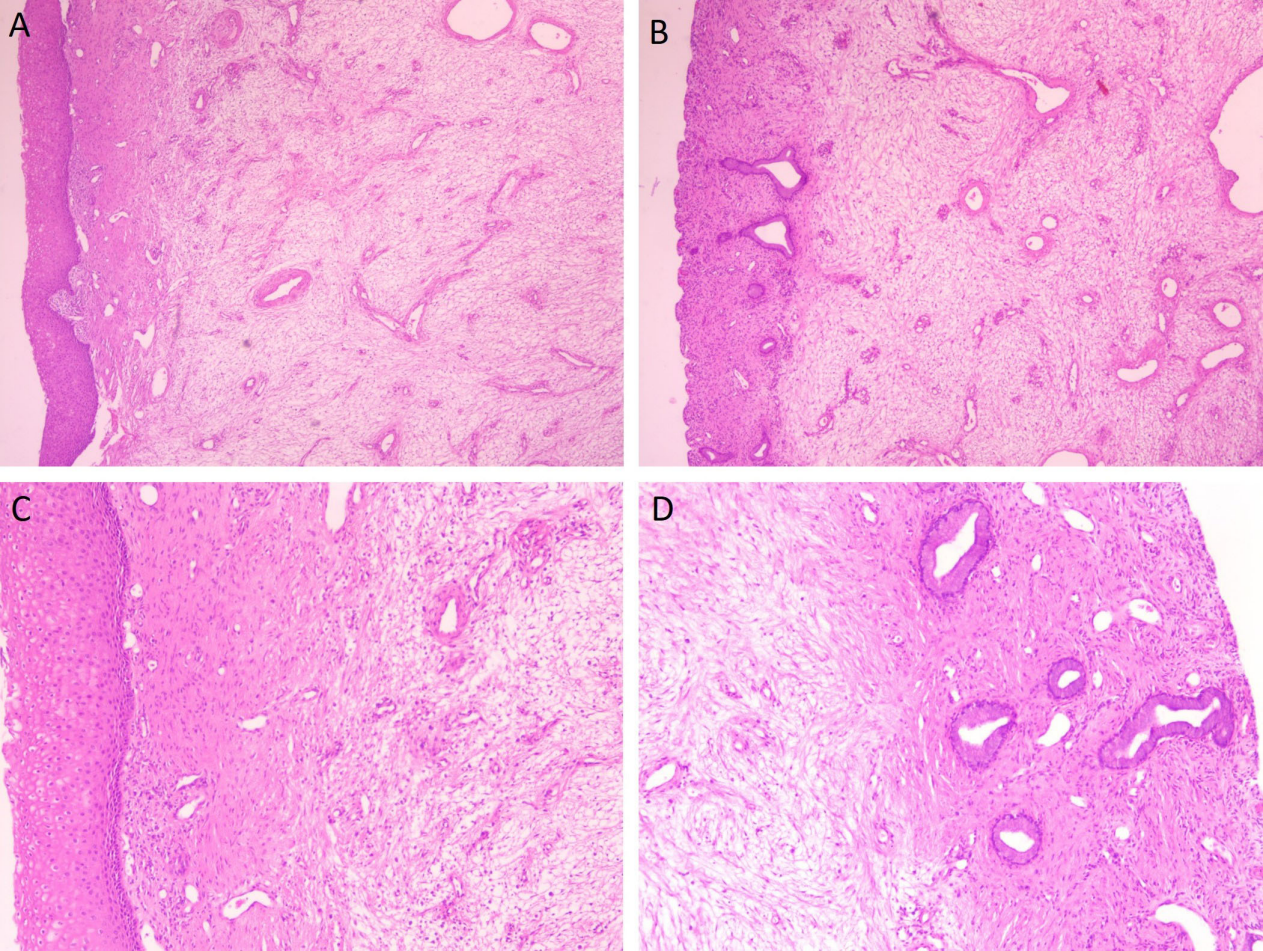
Microscopic features of superficial cervicovaginal myofibroblastoma. [**(A, B)**; 4×] The tumor is composed predominantly of spindle cells arranged in fascicular and whorled patterns, with focal woven or storiform architecture. Collagen fibers and sparse elastic fibers are present between tumor cells. [**(C)**; 10×] Scattered lymphocytes and plasma cells are observed at the tumor periphery. [**(D)**; 10×] Numerous vascular structures with intact vessel walls are present, without evidence of vascular invasion.

At low magnification, the tumor consisted predominantly of spindle-shaped cells with moderate cellularity. The cells were arranged in fascicular and whorled patterns, with focal areas demonstrating a woven or storiform architecture. Collagen fibers and sparse elastic fibers were present between tumor cells, forming a fibrous stromal background (**Figures 3A, B**). The tumor margins were relatively well defined but lacked a true capsule. In the peripheral regions of the lesion, scattered infiltration of chronic inflammatory cells was observed, mainly lymphocytes and plasma cells (**Figure 3C**). In addition, numerous vascular structures were present, predominantly small vessels with intact walls and no evidence of vascular invasion (**Figure 3D**).

**Figure 4:**
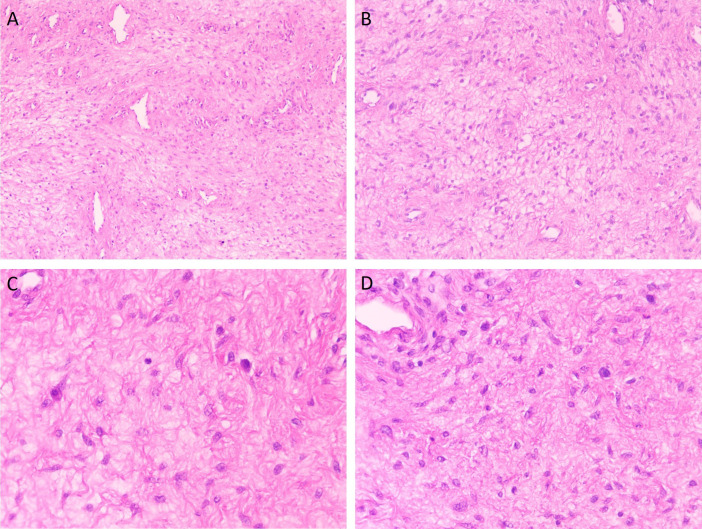
High-magnification microscopic features of superficial cervicovaginal myofibroblastoma. [**(A, B)**; 20×] Tumor cells are spindle-shaped, with pale eosinophilic cytoplasm and oval to spindle-shaped nuclei with smooth, intact nuclear membranes, without obvious necrosis or hemorrhage. [**(C, D)**; 40×] Chromatin demonstrates fine granular distribution, nucleoli are small and inconspicuous, and filamentous structures are visible within the cytoplasm.

At high magnification, tumor cells exhibited spindle-shaped morphology with pale eosinophilic cytoplasm. The nuclei were oval to spindle-shaped with smooth and intact nuclear membranes. Nuclear size was relatively uniform, with minimal atypia and rare mitotic figures, averaging 1–2 mitoses per 50 high-power fields. No areas of tumor necrosis or hemorrhage were identified. Chromatin appeared finely granular, and nucleoli were small and inconspicuous. Filamentous structures were observed within the cytoplasm. Focal mild myxoid degeneration was noted around tumor cells in certain areas (**Figure 4**).

### 2.3 Immunohistochemical testing

Immunohistochemical staining was performed using the EnVision two-step method in accordance with the manufacturer’s instructions. Both cases demonstrated identical immunohistochemical profiles. SMA and DES demonstrated strong cytoplasmic positivity in tumor cells. CD34 was positive in vascular endothelial cells and spindle cells, with weak positivity observed in some tumor areas. Tumor cell nuclei demonstrated diffuse strong positivity for estrogen receptor and progesterone receptor, indicating hormone-responsive characteristics of this tumor (**Table 1**).

**Table 1:** Immunohistochemical staining results in two patients with SCVM.

Case	SMA	Desmin	CD34	ER	PR	S-100	CK	Ki-67
1	+	+	+	+	+	–	–	<3%
2	+	+	Weak +	+	+	–	–	<3%

**Figure 5:**
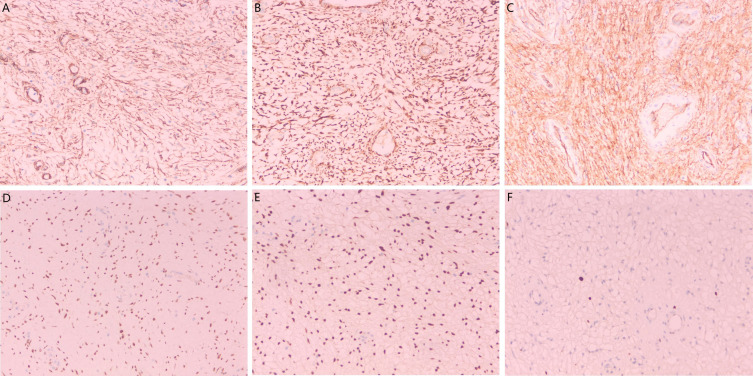
Immunohistochemical findings in superficial cervicovaginal myofibroblastoma. [**(A–F)**; 20×] Tumor cells demonstrate expression of SMA, DES, CD34, ER, PR, and Ki-67, respectively. SMA and DES demonstrate strong cytoplasmic positivity. CD34 is positive in vascular endothelial cells and spindle cells. Tumor cell nuclei demonstrate diffuse strong positivity for ER and PR. The Ki-67 proliferation index is less than 2%.

Positivity for CD34, estrogen receptor, and progesterone receptor excluded the diagnosis of a smooth muscle tumor. Negative staining for S-100 protein ruled out a neurogenic tumor, while negative cytokeratin expression excluded an epithelial tumor. The Ki-67 proliferation index was less than 2% in both cases, indicating low proliferative activity consistent with the benign biological behavior of the tumor (**Figure 5**).

### 2.4 Molecular pathology testing

**Figure 6:**
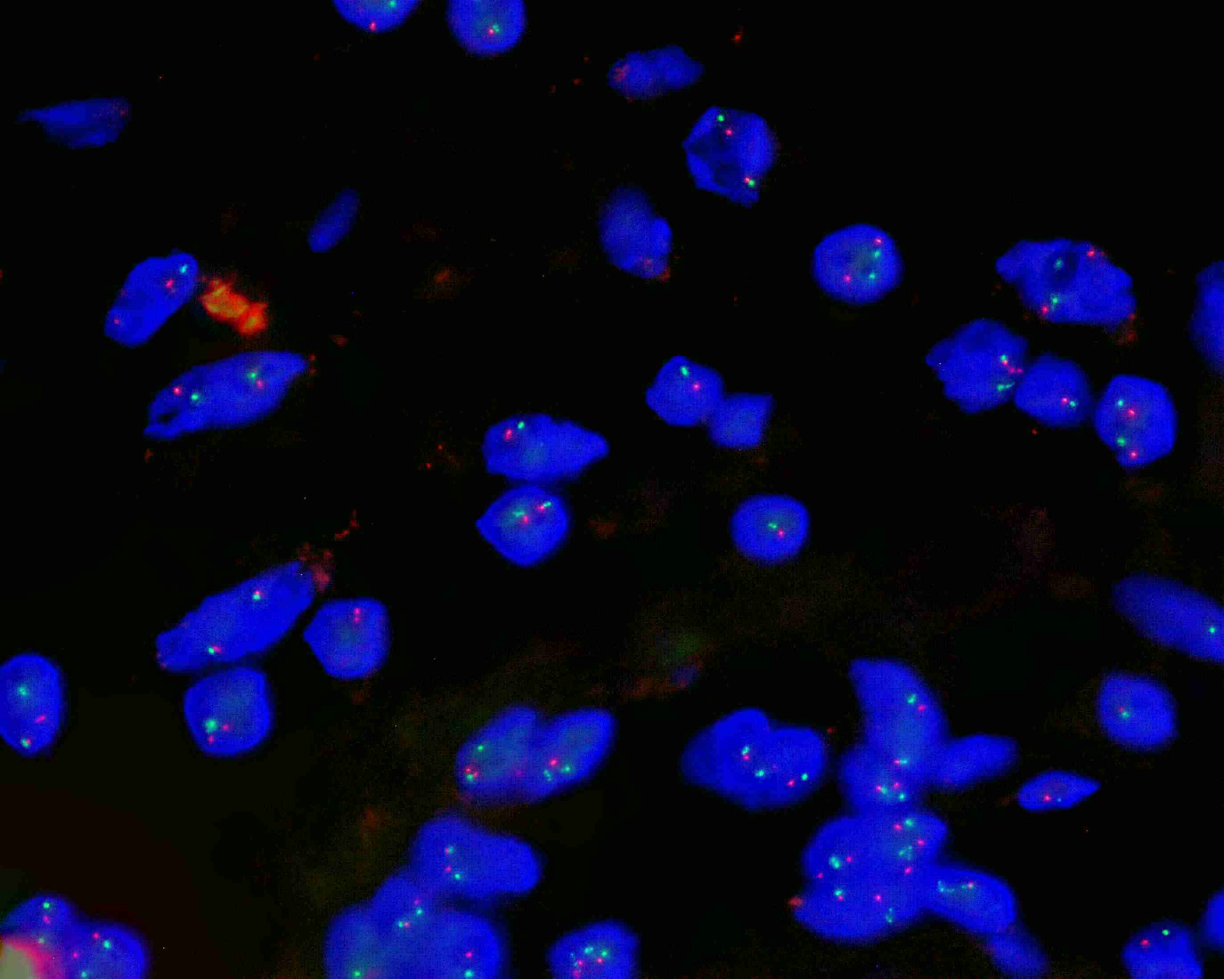
FISH analysis of the *RB1* gene at 13q14 in SCVM: Detection probe: FastProbe *RB1* (13q14)/LAMP1 (13q34) dual-color probe. The orange-red (R) signal represents D13S319 (13q14), and the green (G) signal represents LAMP1 (13q34). The normal negative signal pattern is 2R2G, and the typical positive signal pattern is 1R1G or 1R2G. Test results: A total of 100 cells were counted, with 1R1G cells accounting for 9%, 2R2G cells for 84%, 2R3G cells for 2%, and 3R3G cells for 5%. Test conclusion: This sample demonstrates a negative FISH result for *RB1* (13q14) gene deletion.

To further support the diagnosis and exclude other entities, molecular pathology testing was performed on the specimen obtained from the patient with vaginal bleeding. Fluorescence *in situ* hybridization (FISH) was used to assess deletion of the *RB1* gene, which is frequently deleted within the spectrum of myofibroblastic tumors and serves as a characteristic molecular marker. The analysis demonstrated no deletion of the *RB1* gene at 13q14 (**Figure 6**; 100 cells were counted: 1R1G 9%, 2R2G 84%, 2R3G 2%, 3R3G 5%). Nevertheless, based on the combined assessment of morphological features and immunohistochemical findings, the diagnosis of SCVM was supported.

## 3 Differential diagnosis

### 3.1 Differentiation from fibroepithelial stromal polyp

Fibroepithelial stromal polyp (FSP) is typically covered by an epithelial layer composed of cervical columnar epithelium, squamous epithelium, or squamocolumnar junction epithelium. The epithelial component may demonstrate hyperplasia and squamous metaplasia, with focal erosion and parakeratosis secondary to friction or inflammation. Glandular structures may demonstrate mild hyperplasia or dilatation. The stromal component consists of loose to dense fibrous connective tissue containing fibroblasts and collagen fibers, often accompanied by mild infiltration of lymphocytes and plasma cells. Stromal loosening may occur due to edema, congestion, or myxoid degeneration, without evidence of myogenic differentiation (6). FSP typically lacks a grenz zone, and the lesion often extends to the dermal–epidermal junction (7). In contrast, SCVM is characterized by proliferation of myofibroblasts, thick-walled vessels, and hyalinized collagen. Immunohistochemically, FSP is negative for SMA and DES, although CD34 expression may be present.

### 3.2 Differentiation from schwannoma

Schwannoma arising in the cervicovaginal region is extremely rare and requires differentiation from myofibroblastoma. Schwannoma is composed of Schwann cells and histologically demonstrates characteristic alternating Antoni A and Antoni B areas. Antoni A areas demonstrate densely packed cells arranged in palisades with Verocay bodies, whereas Antoni B areas contain sparsely distributed cells within a myxoid stroma (8). In contrast, myofibroblastoma lacks these distinctive architectural features, with tumor cells more commonly arranged in fascicular and whorled patterns. Immunohistochemical evaluation is essential for distinction, as schwannoma demonstrates strong positivity for S-100 protein and is negative for myogenic markers such as SMA and DES, whereas myofibroblastoma demonstrates the opposite immunophenotype (3).

### 3.3 Angiomyofibroblastoma

Angiomyofibroblastoma most commonly arises in the female vulva, particularly the labia majora, with approximately 10%–15% of cases occurring in the vagina and rare involvement of the cervix and perineum (9). Histologically, the tumor exhibits alternating hypercellular and hypocellular areas. Tumor cells are predominantly plump spindle-shaped or epithelioid, and binucleated or multinucleated cells are frequently observed. A characteristic feature is the perivascular growth pattern surrounding thin-walled blood vessels. Mast cell infiltration may be present within the stroma, and some cases demonstrate variable amounts of mature adipose tissue. CD34 expression is negative or only focally weakly positive (10).

### 3.4 Differentiation from aggressive angiomyxoma

Aggressive angiomyxoma typically occurs in the pelvis and perineum of young to middle-aged women and may involve the vulva and retroperitoneal pelvic space. The tumor often demonstrates deep infiltration into surrounding tissues and may adhere to adjacent structures such as the vaginal wall and rectum. Lesions are usually large, with maximum diameters frequently exceeding 5 cm and reaching up to 14 cm, and present as lobulated masses with ill-defined margins (11). Tumor cells are sparsely distributed within abundant myxoid stroma and display stellate, oval, or short spindle-shaped morphology. Irregularly distributed medium-sized to large thick-walled vessels are present, with hyaline degeneration observed in some vessel walls and perivascular regions (12). Loosely arranged smooth muscle-like cells may be identified around vessels, some of which express SMA, whereas S-100 protein and CD34 are negative (13).

### 3.5 Differentiation from endometrial stromal sarcoma

Endometrial stromal sarcoma is a malignant tumor derived from endometrial stromal cells and accounts for approximately 0.2%–1% of all uterine malignancies. Although tumor cell morphology may resemble that of myofibroblastoma, with spindle-shaped cells, endometrial stromal sarcoma typically demonstrates higher cellularity, increased mitotic activity, and characteristic vascular invasion (14). Immunohistochemically, this tumor demonstrates strong CD10 positivity, with frequent expression of estrogen receptor and progesterone receptor, and may exhibit weak or negative staining for SMA and DES (15). Molecular pathological analysis commonly reveals *JAZF1–SUZ12* or *PHF1–JAZF1* fusion genes in endometrial stromal sarcoma (16), whereas myofibroblastoma generally lacks these specific genetic alterations. In addition, endometrial stromal sarcoma exhibits aggressive biological behavior with a tendency for recurrence and metastasis, resulting in an unfavorable prognosis.

## 4 Treatment and prognosis

### 4.1 Surgical treatment

After a definitive diagnosis was established, both patients underwent local surgical excision of the tumor.

The 41-year-old patient underwent hysteroscopic resection under intravenous anesthesia in the lithotomy position. A vaginal mass measuring approximately 3.5 × 3.0 cm was identified, attached to the posterior cervical wall. The tumor was firm, encapsulated, and irregular in shape. It was excised at its base, and the tumor root (approximately 1.5 × 1.5 cm) was completely removed using a loop electrode. Hemostasis was achieved by electrocoagulation. The remaining cervical mucosa appeared normal.

The 52-year-old patient underwent hysteroscopic excision of a vaginal lesion under intravenous anesthesia. A red lesion measuring approximately 2.5 × 1.5 cm was identified on the upper left vaginal wall and completely removed. No residual tumor was observed, and the vaginal wall and cervix were smooth after excision.

Intraoperative blood loss in both patients was approximately 20–30 mL, and no significant intraoperative complications were observed. Postoperative recovery was uneventful, with no occurrences of fever, infection, or other complications. In the 41-year-old patient, mild vaginal bleeding occurred postoperatively and resolved by the third day. Vaginal packing was removed on postoperative day 7, and satisfactory wound healing was observed.

### 4.2 Adjuvant therapy

Both patients achieved favorable outcomes following surgical resection. Postoperative management included nutritional support and psychological counseling, which contributed to the improvement of overall patient health status. Given that the diagnosis of a rare disease may be associated with psychological distress, targeted psychological support and appropriate guidance were considered particularly important for this patient population.

### 4.3 Follow-up results

**Figure 7:**
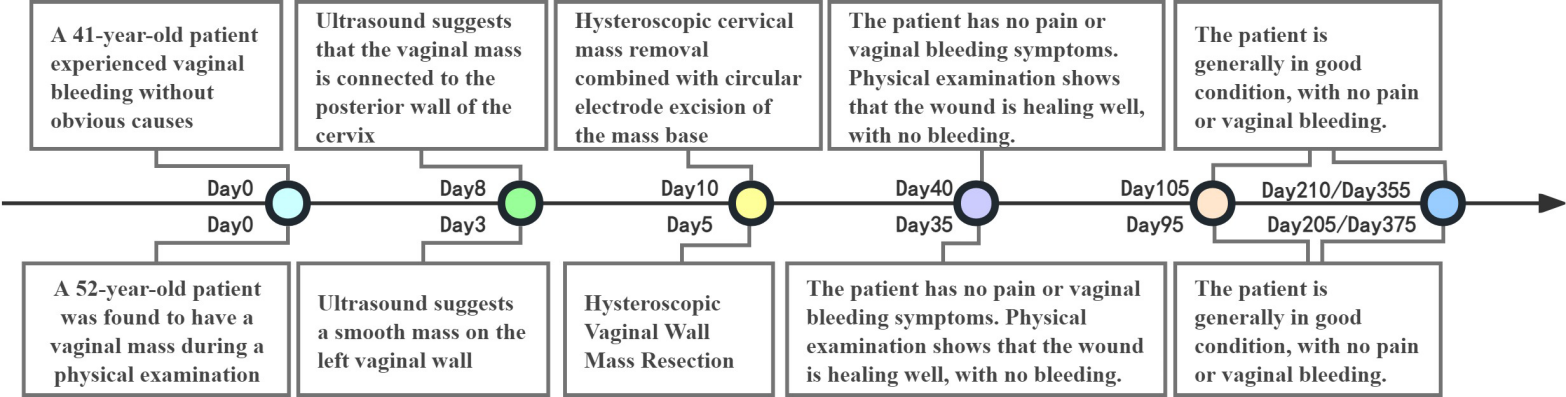
Treatment timeline of the two cases.

Postoperative follow-up at 1 month demonstrated good healing of the surgical site in both patients, with normal cervical morphology and no evident abnormalities. During regular follow-up, neither patient reported clinical symptoms such as irregular vaginal bleeding or pelvic pain. Gynecological examination revealed a smooth cervix without abnormal masses. The 41-year-old patient maintained regular menstrual cycles with normal menstrual volume, while the 52-year-old patient remained in the perimenopausal period without additional discomfort. Overall, quality of life improved markedly (**Figure 7**).

The 52-year-old and 41-year-old patients were hospitalized in 2024 and 2025, respectively. The most recent follow-up was conducted in December 2025 and January 2026, respectively.

## 5 Literature review and discussion

### 5.1 Epidemiological characteristics of SCVM

SCVM is a rare benign mesenchymal tumor that was first described in 2001 (1). A review of the English-language literature published over the past 20 years identified a total of 78 reported cases. The overall incidence of this tumor is extremely low, accounting for less than 0.01% of gynecologic tumors. This entity demonstrates distinct epidemiological characteristics, occurring almost exclusively in the female lower genital tract, with the cervix and upper vagina representing the most frequent sites of involvement. The peak incidence has been reported in perimenopausal women aged 45–55 years, who account for approximately 65% of documented cases (17). This age distribution appears to be closely associated with changes in estrogen levels, as tumor cells consistently express estrogen receptors, indicating a potential role of estrogen in tumor development and progression. Approximately one-quarter of reported patients had a history of tamoxifen use or hormone replacement therapy.

From a geographic perspective, most reported cases originate from Europe and North America, whereas reports from Asia remain relatively limited (4). This disparity may be related to differences in diagnostic techniques, standardization of pathological evaluation, and publication practices. Clinically, approximately 60% of patients present with irregular vaginal bleeding at the time of diagnosis, 25% report increased vaginal discharge, and 15% are identified incidentally during routine physical examination or evaluation for other gynecologic conditions (18). Tumor size demonstrates considerable variability, ranging from 0.8–6.5 cm in diameter, with a mean diameter of 2.8 cm. In the majority of cases, tumor size falls within the range of 2–4 cm (19) (**Table 2** (2, 5, 17–25)).

**Table 2:** Epidemiological characteristics of SCVM reported in the literature.

Author	Number of cases	Age	Location	Lesion size (cm)	Clinical symptoms	Hormone use history	Treatment approach	Prognosis
Laskin et al. (2001) (19)	14	40-74	Vagina 12 cases cervix 2 cases	1-6.5	Asymptomatic 9 cases vaginal bleeding/discharge 2 cases	Hormone replacement therapy 7 cases, tamoxifen 2 cases, oral contraceptives 1 case	Local excision 13 cases	No recurrence 11 cases, lost to follow-up 2 cases, not mentioned 1 case
Ganesan et al. (2004) (20)	11	23-80	Vaginal fornix 2 cases vagina 7 cases, vulva 2 cases	2-4.5	–	Tamoxifen 3 cases	Local lesion excision 10 cases, total hysterectomy 1 case	No recurrence 10 cases, 2 cases lost to follow-up
Stewart et al. (2005) (2)	4	40-71	Vagina 3 cases, cervix 1 case	1.6-4.5	2 cases discovered during surgery for cervical intraepithelial neoplasia	Tamoxifen 1 case	Local excision	No recurrence 3 cases, recurrence 1 case
Wang et al. (2010) (21)	4	47-63	Vagina 4 cases	1.5-3.7	1 case with vaginal discharge, 1 case discovered due to vaginal infection, 2 cases detected during physical examination	Tamoxifen 1 case	Local lesion excision	No recurrence 4 cases
Magro et al. (2012) (22)	10	44-77	Vagina 8 cases, vulva 2 cases	0.4-3	–	1 case with prior hormone use, 1 case with long-term oral contraceptive use	Local excision	No recurrence 6 cases, lost to follow-up 4 cases
Smith et al. (2017) (5)	1	73	Upper vagina	4.7	Painless vaginal mass	–	–	–
Abdelaziz et al. (2017) (23)	1	45	Anterior lip of the cervix	5	Menorrhagia	–	Total hysterectomy	–
Atinga et al. (2018) (24)	1	50	Posterior vaginal fornix	1.6	Vaginal bleeding	Not used	Total hysterectomy and bilateral salpingo-oophorectomy	No recurrence
Chen et al. (2018) (25)	12	35-51	Vagina 9 cases, cervix 3 cases	0.8~3.6	Vaginal bleeding 8 cases, asymptomatic 4 cases	–	Local excision 11 cases, total hysterectomy 1 case	No recurrence 12 cases
Tajiri et al. (2021) (17)	8	26-74	Vagina 5 cases, cervix 1 case, vulva 2 cases	1-10	–	–	–	–
Zhang et al. (2022) (18)	15	34-73	Vagina 12 cases, cervix 1 case, vulva 2 cases	0.4-6.5	1 case with vaginal bleeding, 14 cases with painless mass	–	Total hysterectomy 10 cases, uterus plus bilateral salpingectomy 2 cases	No recurrence 10 cases, lost to follow-up 5 cases

### 5.2 Pathological characteristics of SCVM

A systematic review of the literature indicates relatively consistent pathological characteristics for this tumor. On gross examination, SCVM typically presents as a solitary nodular or polypoid lesion, most commonly measuring 1–4 cm in diameter (26). The surface is usually smooth or mildly lobulated, and the lesion has a firm consistency. On cut surface, the tumor appears grayish white to pale yellow with a collagenous texture. In most cases, a complete capsule is absent, although the lesion generally demonstrates relatively clear, albeit irregular, borders. The tumor tissue is typically uniform, with occasional focal hemorrhage or necrosis, whereas extensive necrosis and cystic degeneration are rare (21). Microscopically, the tumor is composed predominantly of spindle-shaped cells arranged in fascicular, woven, or focal whorled patterns. The stroma contains abundant collagen fibers. Tumor cells exhibit pale eosinophilic cytoplasm, oval to spindle-shaped nuclei, evenly distributed chromatin, and inconspicuous nucleoli (20).

Immunohistochemical analysis plays a critical role in establishing the diagnosis. Review of published cases indicates that most tumors demonstrate strong positivity for vimentin, SMA, and DES, with CD34 generally positive, whereas S-100 protein and cytokeratin are negative. The Ki-67 proliferation index is typically below 5%, reflecting low proliferative activity (**Table 3** (2, 5, 18–25)) (27). Some studies have reported histogenetic similarities between SCVM and mammary-type myofibroblastoma, with FISH demonstrating RB1 gene deletion at 13q14 (22). In the two cases presented in this report, FISH analysis for RB1 gene deletion at 13q14 was performed in one patient with vaginal bleeding and yielded negative results. Reports involving molecular analysis remain limited, and accumulation of additional cases is required to further clarify the molecular characteristics of this tumor.

**Table 3:** Summary of immunohistochemical findings of SCVM reported in the literature.

Author	Number of cases	Vimentin	DES	SMA	ER	PR	CD34	MSA	Caldesmon	S-100	Ki-67	Mitotic figures
Laskin et al. (2001) (19)	14	5/5	13/13	5/11	10/10	10/10	11/13	2/8	–	–	–	< 2/10 HPF
Ganesan et al. (2004) (20)	11	11/11	9/11	–	9/11	–	6/12	–	0/11	0/11	–	< 1/10 HPF
Stewart et al. (2005) (2)	4	4/4	4/4	4/4	4/4	4/4	3/4	–	4/4	0/4	–	–
Wang et al. (2010) (21)	4	4/4	4/4	0/4	4/4	4/4	4/4	–	0/4	0/4	–	No mitotic figures observed
Magro et al. (2012) (22)	10	10/10	10/10	0/10	8/8	8/8	7/8	–	–	–	–	–
Smith et al. (2017) (5)	1	1/1	1/1	1/1	1/1	1/1	0/1	0/1	0/1	0/1	–	
Abdelaziz et al. (2017) (23)	1	–	1/1	1/1 (partial)	–	–	1/1	–	–	–	–	–
Atinga et al. (2018) (24)	1	–	1/1	0/1	1/1	1/1	1/1	–	–	0/1	1 case < 2%	–
Chen et al. (2018) (25)	12	12/12	10/12	4/12	10/10	10/12	9/12	–	–	0/12	12 cases < 2%	–
Zhang et al. (2022) (18)	15	12/12	14/15	4/14	15/15	13/14	8/15	–	2/8	–	15 cases < 5% 13 cases < 3%	–

### 5.3 Treatment strategy for SCVM

Analysis of the available literature indicates that the treatment strategy for SCVM is well established and associated with an excellent prognosis. Complete surgical excision remains the standard treatment, and most patients achieve favorable outcomes with tumor removal alone. Over the past five years, minimally invasive techniques in gynecologic tumor management have advanced substantially, with laparoscopy and hysteroscopy being applied in more than 70% of procedures for benign cervical lesions (28). The surgical approach should be individualized based on tumor location, size, and patient-specific factors. Small lesions located on the cervical surface may be treated by local excision, whereas larger or more deeply situated tumors may require cervical conization. Surgical resection should aim to achieve negative margins whenever feasible, with a generally recommended margin of 0.5–1.0 cm from the tumor boundary. Intraoperative frozen section evaluation may assist in confirming complete excision and reducing the risk of residual disease (28).

As a benign tumor, SCVM does not typically require adjuvant radiotherapy or chemotherapy following surgery. Among the 78 reported cases in the English-language literature, recurrence and distant metastasis after complete excision have been exceedingly rare, and the reported 5-year survival rate is 100% (21). Postoperative follow-up generally includes regular gynecological examination and imaging assessment, commonly recommended at 1 month, 3 months, and 6 months after surgery, followed by annual evaluation. In younger patients with reproductive requirements, conservative surgical management has not been associated with impairment of reproductive function (29). Although recurrence is extremely uncommon, long-term follow up is still advised. In rare cases in which postoperative pathological assessment indicates positive margins, repeat surgical excision should be considered, or close observation with individualized management strategies based on tumor growth characteristics may be adopted (30).

### 5.4 The potential role of estrogen hormone in the pathogenesis of SCVM

The biological effects of estrogen are primarily mediated through estrogen receptors (ER), which include nuclear receptors (ERα and ERβ) and membrane receptors (GPER). Among these, genomic effects mediated by nuclear receptors represent the primary pathway involved in tumorigenesis (31).

Approximately one-third of patients with SCVM have a history of tamoxifen use or exposure to hormone replacement therapy (HRT). As a selective estrogen receptor modulator, tamoxifen exerts mild estrogen-like effects and may induce the differentiation of primitive mesenchymal cells in the cervix and vagina into myofibroblasts via activation of ER signaling, thereby contributing to tumorigenesis. In addition, estrogen may enhance the contractile and secretory functions of myofibroblasts by regulating their phenotypic characteristics, promoting tumor formation and stability. These mechanisms are consistent with the histopathological features of SCVM, including disorganized spindle-shaped myofibroblasts and stromal myxoid change or collagenization (19).

Multiple clinical studies have demonstrated that SCVM tumor cells generally exhibit diffuse positive expression of ER and progesterone receptor (PR) (32). In the collected cases, the positive expression rates of ER and PR were 98% (62/64) and 94% (51/54), respectively (**Table 3** (2, 5, 18–25)). These findings indicate that ER/PR positivity is a typical immunophenotypic feature of SCVM. This expression pattern is consistent in tumors arising in both the cervix and vagina, providing molecular evidence supporting the role of estrogen in the pathogenesis of SCVM.

## 6 Conclusion

SCVM is an extremely rare benign mesenchymal tumor within the spectrum of gynecologic neoplasms. Based on detailed clinicopathological analysis of two patients and a systematic review of 78 previously reported cases, the diagnostic features and key points for differential diagnosis of SCVM were further clarified. The precise histogenesis of SCVM remains unclear; however, it is generally considered to originate from specialized stromal cells of the female lower genital tract. Several reports have indicated an association with tamoxifen use, whereas evidence supporting a viral etiology has not been identified. Imaging studies typically demonstrate localized soft tissue masses involving the cervix or vagina.

Experience derived from the successful diagnosis and treatment of these cases indicates that SCVM should be considered when unexplained neoplastic lesions are identified in the cervix or vagina, in order to reduce the risk of misdiagnosis related to limited disease awareness. Complete surgical excision remains the treatment of choice, and when negative margins are achieved, the prognosis is favorable with an extremely low risk of recurrence. To date, only one case of local recurrence has been reported, occurring 9 years after local excision. Accurate pathological diagnosis relies on expertise in morphological assessment and immunohistochemical interpretation, and a multidisciplinary collaborative approach may further enhance diagnostic accuracy. This report expands the existing clinical case database of SCVM, provides a valuable reference for future research, and underscores the importance of individualized treatment strategies. When similar rare cases are encountered in clinical practice, the diagnostic and therapeutic approaches described herein may serve as useful references.

## Data Availability

The raw data supporting the conclusions of this article will be made available by the authors, without undue reservation.
